# Dr. Koecker on the Diseases of the Gums

**Published:** 1842-06

**Authors:** 


*Dr. Koecker on the Diseases of the Gums.*—In the last number of our Journal, which contained a highly interesting paper on the diseases of the gums, by Dr. H. H. Hayden, we stated that Dr. Koecker's views upon the same subject would appear in the regular course of the publication of his "Principles of Dental Surgery," in the library part of our present number. They will be found under the head of "the devastating process," commencing on page 144, to which we beg leave to refer our readers.

